# Coregistration of EEG and eye-tracking in infants and developing populations

**DOI:** 10.3758/s13414-024-02857-y

**Published:** 2024-02-22

**Authors:** Louisa Kulke

**Affiliations:** Department of Developmental Psychology with Educational Psychology, University of Bremen, Hochschulring 18, 28359 Bremen, Germany

**Keywords:** Co-registration, EEG, Eye-tracking, Infancy, Gaze-contingency

## Abstract

Infants cannot be instructed where to look; therefore, infant researchers rely on observation of their participant’s gaze to make inferences about their cognitive processes. They therefore started studying infant attention in the real world from early on. Developmental researchers were early adopters of methods combining observations of gaze and behaviour with electroencephalography (EEG) to study attention and other cognitive functions. However, the direct combination of eye-tracking methods and EEG to test infants is still rare, as it includes specific challenges. The current article reviews the development of co-registration research in infancy. It points out specific challenges of co-registration in infant research and suggests ways to overcome them. It ends with recommendations for implementing the co-registration of EEG and eye-tracking in infant research to maximise the benefits of the two measures and their combination and to orient on Open Science principles while doing so. In summary, this work shows that the co-registration of EEG and eye-tracking in infant research can be beneficial to studying natural and real-world behaviour despite its challenges.

## Introduction: History of controlled laboratory paradigms

In the real world, people can shift their attention relatively freely and direct it towards objects they are curious about. However, for a long time, neuroscientific research has instructed participants where to look in controlled laboratory studies. Eye movements have been avoided as they can induce large artefacts in neuroimaging data recording (Corby & Kopell, 1972; Croft & Barry, 2000; Joyce et al., 2004; Luck, 2005). Therefore, adult participants were instructed not to move their eyes when neural mechanisms of attention were studied (e.g., Anllo-Vento & Hillyard, 1996; Eimer et al., 2002, 2005; Martinez et al., 1999; Praamstra & Oostenveld, 2003; Shomstein et al., 2012; Yamaguchi et al., 1994, 1995) even though the suppression of eye movements can lead to less natural brain responses (Kulke, 2019; Kulke, Atkinson, & Braddick, 2016a; Perry & Zeki, 2000).

However, it is not possible to instruct infants and preverbal populations on how to behave and where to look. Instead, infant researchers have observed infants’ natural eye movements. Very early studies usually relied on an experienced ‘blind’ adult observer judging the time and direction of the infant’s eye movements and making a manual response (e.g., Atkinson et al., 1992; Richards, 2005) or frame-by-frame video analysis of eye movements (e.g., Butcher et al., 2000; Elsabbagh et al., 2013; Hood & Atkinson, 1993; Hunnius & Geuze, 2004; Hunnius et al., 2008; Matsuzawa & Shimojo, 1997).

**Fig. 1:**
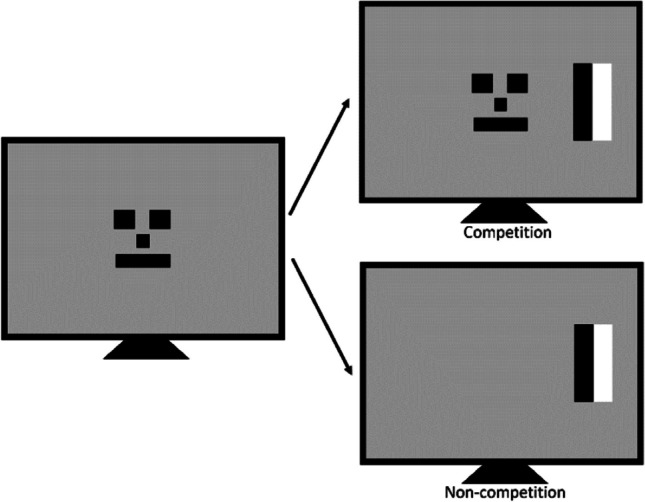
The Fixation Shift paradigm (Atkinson et al., 1988, 1992; Hood & Atkinson, 1993) is an early attention shift paradigm that presents infants with a central stimulus (e.g., a face) followed by a peripheral target that is either competing for attention with the first stimulus (Competition condition) or not (Non-competition condition). This paradigm was the first paradigm in which infant researchers co-registered eye-tracking with EEG to study attention development (Kulke et al., 2016b)

Many classic studies used infants’ natural gaze behaviour to develop eye-movement-based paradigms to measure infant development, for example, attention development (Atkinson et al., 1988, 1992; Hood & Atkinson, 1993; Kulke et al., 2015), social development (e.g., Kulke & Rakoczy, 2018; Low & Watts, 2013; Southgate et al., 2007; Surian & Geraci, 2012) and moral development (Hamlin et al., 2007). These gaze-based paradigms include *habituation* paradigms, in which an object or situation is presented to an infant, and their gaze towards it decreases during the repetitive presentation but increases again when a novel stimulus is presented (Baillargeon et al., 1985; Cohen, 1976). In particular, *violation of expectation* looking-time paradigms (Onishi & Baillargeon, 2005) make use of the natural behaviour of infants to look longer at an event or object that violates their expectation (for an overview, see Margoni, Surian, & Baillargeon, 2023). These paradigms allow us to determine if infants can detect unusual events. *Preferential looking* paradigms (Johnson et al., 1991; Spelke, 1985) have been used from birth on and even before birth to determine if infants have a preference for certain stimuli*. Anticipatory looking* paradigms measure if infants look in anticipation to a location where they expect something interesting to appear (Bower et al., 1971; Bower, 1974; Kulke, Reiß, Krist, & Rakoczy, 2018a; Senju et al., 2009; Southgate et al., 2007). *Gaze shift* paradigms such as the *Fixation Shift Paradigm* or *Gap/Overlap Paradigm* have been used to measure early attention (Atkinson & Hood, 1993; Atkinson et al., 1988, 1992; Hood & Atkinson, 1993). In this paradigm (Fig. 1), infants are presented with a central fixation stimulus followed by either one peripheral target (non-competition condition) or a peripheral and a central target that are competing for attention (competition condition) (Atkinson et al., 1988, 1992; Hood & Atkinson, 1993). Early pioneering work demonstrated that very young newborns have difficulty in disengaging attention when two targets are competing for attention, while they are able to shift attention to single targets in the non-competition condition (Atkinson et al., 1988, 1992; Braddick & Atkinson, 2011; Hood & Atkinson, 1993). Even before modern eye-tracking tools were used in laboratories, these paradigms allowed us to measure overt attention in typical and atypical populations (Atkinson et al., 2003, 2008; Elsabbagh et al., 2009; Gliga et al., 2014; HIE, Mercuri et al., 1997, 1999). Research on attention of infants who had one cortical hemisphere removed (hemispherectomised children) (Braddick et al., 1992) further inspired theories about the neural mechanisms underlying attention development by Braddick and Atkinson (2011) and Johnson (2001) before co-registration of EEG and eye-tracking was possible.

Paradigms measuring natural eye-movement behaviour as overt attention, therefore, flourished in infant research. In the meantime, most neuropsychological laboratory research on adults instructed participants to keep their fixation still to avoid eye-movement artefacts (Corby & Kopell, 1972; Croft & Barry, 2000; Joyce et al., 2004; Luck, 2005), although some examples also investigated free viewing and reading in adults (Csibra et al., 1997; Dimigen et al., 2011; Huber-Huber et al., 2016, 2021; Kulke, 2019; Kulke et al., 2016a, 2020; Kulke, Brümmer, Pooresmaeili, & Schacht, 2021a, 2022a).

Infant research therefore started early on to investigate attention with natural eye movements (e.g., Atkinson et al., 1988, 1992; Hood & Atkinson, 1993; Kulke et al., 2015) and in the real world (e.g., Aslin, 2009; Smith et al., 2015; Yoshida & Smith, 2008). Although the infant’s natural eye movements offered invaluable information, the cognitive mechanisms underlying their gaze behaviour were still unrevealed because of infants’ limited verbal skills. Thus, developmental scientists quickly developed an interest in measuring neural processes to identify what infants can perceive and which cognitive processes underlie their behaviour. EEG is considered a particularly useful method to study cognitive processes in infants (DeBoer et al., 2007; Johnson et al., 2001; Luck & Kappenman, 2011; Thomas & Casey, 2003). EEG had already been implemented as a reliable measure of neural responses in infants (for a review, see e.g., Braddick & Atkinson, 2011; MCculloch, 2013). Visually Evoked Potentials (VEPs) can be used from birth onwards (Fielder et al., 1983; Lee et al., 2012a), and their development across ages had been mapped (Barnet et al., 1980a; Fielder et al., 1983; Lee et al., 2012a, 2012b; McCulloch, 2007; McCulloch & Skarf, 1991; Nelson & McCleery, 2008). Attention affects the amplitudes of visually evoked potentials in infants (de Haan, 2007; Reynolds & Richards, 2005; Richards, 2000, 2005) and attentional processing in infants is furthermore reflected in a central negativity (de Haan, 2007; Richards, 2003), showing that attentional modulations could be detected using EEG.

Infant researchers, therefore, started early on to combine gaze measures and EEG. Even before eye-tracking was implemented in infant research, early infant work allowed infants move their eyes freely while measuring EEG (e.g., Csibra et al., 1998). Csibra et al. (1998) measured fixation shifts in infants and simultaneously recorded EEG. They computed event-related potentials as well as fixation-related potentials by determining eye movements from ocular electrodes. This allowed them to gain a picture of attention in the first year of life. Additionally, researchers started to combine video recordings of gaze with EEG to measure novelty preference (e.g., Reynolds et al., 2010). In 2010, a combination of EEG and behavioural measures was recommended in infant research, although studies were rare (Reynolds & Guy, 2012).

**Fig. 2:**
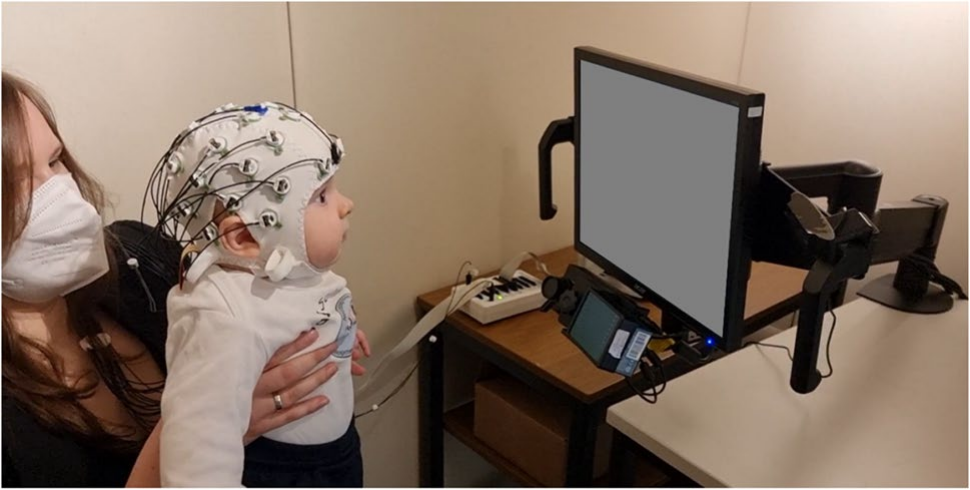
Infant co-registration set up: The infant is seated on the caregiver’s lap, wears an EEG cap, and is presented with stimuli while an eye-tracker (under the monitor) tracks the gaze

When more reliable eye-tracking methods were available, the classic infant attention paradigms, which previously used manual gaze coding or video recording of gaze, were combined with eye-tracking to automatically process gaze data (e.g., Kulke et al., 2015). This automatization made it possible to combine eye-tracking and EEG in infants (Fig. 2). To implement this new co-registration of methods, as a first step, classic infant attention paradigms were combined with eye-tracking and EEG and tested with adult populations to determine if this combination was possible, which was the case (Kulke et al., 2016a). Kulke et al. (2016b) were the first to implement co-registered EEG and eye-tracking in infants to study attention. They used the Fixation Shift Paradigm to measure attention shifts in infants between 1 and 8 months old with simultaneous eye-tracking and EEG, showing that attention shift latency decreased with age, coinciding with a restructuring of neural responses. The findings could add to the understanding of neural underpinnings of attention from this earlier work (Braddick & Atkinson, 2011). Only very few EEG and eye-tracking co-registration studies have been conducted in infants since then. Bache et al. (2017) combined eye-tracking and EEG in 10-month-old infants to investigate differences in the processing of continuous compared to interrupted videos, showing effects on both gaze and rhythmic neural brain activity. Monroy et al. (2019) combined EEG and eye-tracking to study the learning of action sequences in 8- to 11-month-olds. Tan et al. (2022) simultaneously recorded EEG and eye movements in 5-month-olds in response to audio/visual speech, showing a relation between infants’ gaze to the mouth of a speaker and their cortical tracking of visual speech, as well as developments until adulthood. Eye-tracking can also be used as a gaze control mechanism for EEG studies with infants to ensure that they fixate on the stimuli of interest, which can improve data quality for 3- to 4-month-olds (Ahtola et al., 2017).

Most recently, infants’ and young childrens’ gaze (measured with web-cam-based tracking) and EEG have simultaneously been recorded during live interactions with confederates during a waiting-room paradigm (Kulke, Ertuğrul, & Reyentanz, 2022b; Kulke et al., 2023; Kulke, Ertuğrul, Reyentanz, & Thomas, 2021b). This research was, for the first time, able to show that infants avoid staring at strangers as indicated by their gaze, even though they are interested in them, as indicated by their EEG alpha power (Kulke et al., 2023, Kulke, Ertuğrul, et al., 2021b). It furthermore shows that the combination of EEG and eye-movement measures in real-life situations can be particularly useful, as behaviour in live interactions differs from behaviour towards videos or pictures. Co-registration of EEG and eye-tracking in infants in real-world settings is therefore a useful option to investigate new hypotheses in infant research (Kulke et al., 2023).

However, co-registration is still rare in infants due to its methodological challenges, which can lead to data loss even if co-registrations are desired (e.g., Köster et al., 2021). Instead, studies have used eye-tracking and EEG paradigms subsequently instead of simultaneously (Falck-Ytter et al., 2021; Köster et al., 2021; Martin & Becker, 2018) or tested older children, for example, from 8 to 12 years of age (Vettori, Dzhelyova, et al., 2020a, Vettori, Van der Donck, et al., 2020b), 3 to 5 years of age (Cowell & Decety, 2015), or 6 years of age and over (Langer et al., 2017).

## Opportunities for co-registered EEG and eye-tracking in infants

In the following sections, opportunities for and challenges of co-registering EEG and eye-tracking in infants are reviewed.

### Natural eye movements

The co-registration of EEG and eye-tracking in infants brings some new advantages. The combination can be used in very young infants and in preverbal populations (Kulke, 2015). It allows infants to move their eyes freely, which is required due to their lack of understanding of instructions. It, therefore, permits a combination of classic gaze-based infant paradigms with neural measures, allowing a deeper understanding of the mechanisms underlying behavioural and cognitive mechanisms in infants.

Gaze-contingent eye-tracking furthermore allows researchers to adjust the research procedure to infants’ gaze patterns during the test session (Kulke, 2015). Gaze-contingent programming of experiments can also save time if the program automatically continues when the infant is looking, and no additional control by the experimenter is necessary (Kulke et al., 2016b).

### Fixation control

Eye-tracking can be used for gaze control in neuroscientific studies (e.g., Domínguez-Martínez et al., 2015). As infants cannot be instructed to stay still, this helps ensure that EEG data are only analysed when infants are fixating on the target of interest (Ahtola et al., 2017). It can also help exclude the possibility that findings in EEG data of infants are related to microsaccades (Köster, 2016).

### Improved artefact control

Co-registered gaze data can also improve artefact identification in the infants’ data. As eye movements of infants cannot be avoided through instructions, their measurement makes it possible to improve artefact rejection using modern toolboxes (Dimigen et al., 2011), which can be useful in infant populations (Kulke, 2015). These toolboxes make use of the co-registered eye-tracking data to detect instances with eye artefacts and improve independent component analysis (ICA) quality (Dimigen et al., 2011). Another example of avoiding eye-movement artefacts is analysing EEG data only during time periods when no eye movements occurred, with eye-tracking data being used to exclude trials with eye movements (Kulke, 2015).

Filters should be avoided as they can distort even eye-movement-free data in datasets with frequent eye movements (Kulke & Kulke, 2020). This occurs because eye-movements induce large voltage changes, similar to a rectangular pulse. If a high-pass filter is applied to such a pulse in the frequency domain, the lower frequencies are blocked or weakened. If the frequency-domain data are transformed back to the time-domain using a Fast Fourier Transformation, the voltage changes induced by the lack of low-frequency oscillations due to the high-pass filter affects slopes in the vicinity of the rectangular pulse (i.e., eye movement). This results in a filter error revealing itself as a higher slope of the opposite polarity before and after the eye movement (Kulke & Kulke, 2020).

### Studying saccade-related processes

Another application is the investigation of saccade-related potentials. However, it should be noted that this is also possible without the use of eye-tracking, as even before sufficient infant-friendly eye-trackers were on the market, researchers already succeeded in using ocular electrodes to measure horizontal eye movements simultaneously with EEG in infants (Csibra et al., 1998).

### Conceptual advantages

In infant research, gaze has been used as an indicator of attention for several decades (Atkinson et al., 1988, 1992; Hood & Atkinson, 1993; Kulke et al., 2015). And although gaze and attention can overlap, they can also differ. For example, infants can shift attention covertly (Richards, 2000, 2005). This may be one of the reasons why infant studies that use gaze measures sometimes show mixed findings. For example, if infants look longer at a stimulus, this is sometimes interpreted as preference for novelty and sometimes as preference for familiarity (Houston-Price & Nakai, 2004; Wetherford & Cohen, 1973). Similarly, studies demonstrating Theory of Mind in infants (Senju et al., 2009; Southgate et al., 2007) could recently not be replicated (Kulke et al., 2019; Kulke & Rakoczy, 2018; Kulke, Reiß, et al., 2018a, Kulke, von Duhn, Schneider, & Rakoczy, 2018b; Schuwerk et al., 2018). This does not necessarily mean that infants are incapable of Theory of Mind, but instead might reflect that the gaze measure is not sufficient to detect underlying cognitive processes as gaze is the result of different conflicting cognitive processes such as overt attention conflicting with social inhibition of gaze (Kulke, von Duhn, et al., 2018b; Poulin-Dubois et al., 2018). In fact, recent research has demonstrated that gaze patterns and covert EEG measures of attentiveness can in fact diverge in infants (Kulke et al., 2023). The co-registration of EEG and eye-tracking therefore provides new opportunities to investigate attention in infants and provides a more comprehensive understanding of which cognitive processes underlie the observed gaze patterns.

## Challenges of co-registered EEG and eye-tracking in infants

Using either EEG or eye-tracking on its own in infants is already challenging. When combined, some difficulties can be reduced (e.g., eye-tracking data can be used to improve artefact removal and gaze-contingency can ensure that participants are fixating the stimuli of interest, see section *Opportunities of co-registered EEG and eye-tracking in infants*) while other difficulties add up (Kulke, 2015).

### Challenges with EEG in infants

Challenges with EEG in infants include challenges *during the preparation of the EEG system*. Infants have a limited attention span. Preparing the EEG system takes some time, for example, determining the correct cap size, attaching electrodes, and filling electrodes with gel/liquid. The time for preparing the EEG is deduced from the time that the infant can take part in the study. Furthermore, infants may not like putting on a hat or cap, and thus may respond negatively. Distraction, for example, with interesting toys, may help, as well as creating a positive and comfortable atmosphere for infants and their caregivers. Infants also may not like the feeling of gel/liquid in the electrodes on their heads. It can be useful to warm the gel/liquid up to body temperature before applying it to the head. Additionally, the experimenters need to be particularly careful when placing electrodes over young infants’ scalps, as the risk of injury is higher.

Further challenges arise *during testing*. Infants move freely, which may lead to motion artefacts. If infants are seated on their caregivers’ lap during the testing procedure, it may be useful to instruct the caregivers to stay still (Kulke et al., 2015). Infants are only attentive for a short time, meaning that fewer trials can be completed by them. This may pose a problem as EEG data becomes more reliable when averaged across many trials.

There are furthermore challenges during the *analysis* of infant EEG data. Infant EEG studies have higher attrition rates than infant studies without EEG (Stets et al., 2012). Templates and pipelines for artefact removal are often based on adult participants, and adult head models/scans may be used, which are not fully transferable to infants. Therefore, special infant algorithms need to be developed for ICA (Haresign et al., 2021). It can be useful to apply median threshold procedures for outlier detection in the EEG signal if the data are more noisy (Kulke et al., 2015).

### Challenges with eye-tracking in infants

Although eye-tracking is suitable for developing populations (for a review, see Gredebäck et al., 2009), eye-trackers are often developed for adult populations. In particular, wearable mobile eye-trackers are often developed for adult head sizes, making them unsuitable for infants. Therefore, the accuracy and usability for infants can be challenging (e.g., Morgante et al., 2012). Infants still have more reflective eyes, making it difficult to get a clear eye-tracking signal (Kulke et al., 2015). The signal may be improved by using infant-friendly lenses, adjusting the infant’s position, or using neutral density filters (Kulke et al., 2015). Infants are not attentive for a long time, making it desirable to keep calibrations particularly engaging, for example by using videos or animations short (Gredebäck et al., 2009) or even completely omitting them (Kulke et al., 2015). Infants’ vision is less sharp, meaning that small calibration targets cannot be seen by them. Furthermore, stimuli need to be sufficiently large and have high contrast for young children to see.

### Challenges combining EEG and eye-tracking in infants

When combining EEG and eye-tracking in infants, some of the problems listed above add up, leading to even higher attrition rates (Kulke, 2015, Kulke et al., 2016b). As infant participants are more difficult to recruit than typical adult student populations, with infant studies often being under-powered (Bell & Cuevas, 2012; Frank et al., 2017), the exclusion of participants is particularly dramatic. A solution can be to develop paradigms where data can also be used if only EEG or eye-tracking is recorded, although this may not always be practical or desirable, as eye movements do not always reflect cognitive processes (Foulsham et al., 2011; Kulke, Ertuğrul, et al., 2021a; Laidlaw et al., 2011) and the combination may lead to novel findings regarding differences between visual and neural processing (Kulke, Ertuğrul, et al., 2022b, Kulke et al., 2023, Kulke, Ertuğrul, et al., 2021b).

Another challenge that may be particularly pronounced in infant research is the alignment of EEG and eye-tracking data. In infant research, eye-trackers with low sampling rates are commonly used, as they were found to be useful to avoid data loss. However, when co-registering EEG and eye-tracking, the two datasets need to be aligned, for which timing plays a crucial role, which may be easier when eye-trackers have a higher sampling rate. As delays can be a common problem for EEG systems (e.g., Electrical Geodesics, 2014), the timing of the systems should be measured for the respective experimental set-up (Kulke, 2015).

Even if the specific sections of data that are analysed do not include eye movements, filters can induce artefacts in data that contain eye movements, affecting eye-movement-free periods as well (Kulke & Kulke, 2020). This may be particularly challenging in infants, where eye-movement latencies vary more than in adults (e.g., Kulke et al., 2015), making them less predictable. Researchers should be aware that ICA does not reliably remove all artefacts. In particular, ICA can be challenging in infants because fewer electrodes can be filled with gel in infants and because ICA templates are not ideal for infants.

Specific challenges arise when mobile EEG and eye-tracking are combined. As standard wearable mobile eye-trackers were developed for adults and cannot be worn by infants, the signal needs to be aligned with camera recordings for live interaction studies (Kulke, Ertuğrul, et al., 2021b, Kulke, Ertuğrul, et al., 2022a). These have a lower temporal resolution than high-frequency eye-trackers; therefore, events may fall between two samples recorded by the camera.

### Recommendations

To overcome the specific challenges posed by combining EEG and eye-tracking in infant populations, some steps may help.

*Time* is of the essence when testing infants. As many steps as possible should be prepared before the infant arrives. Parents might be asked for the head circumference in advance so that researchers can prepare the cap before the participant arrives, but it should be noted that parents may not always correctly measure the size, which in turn leads to even more preparation efforts. The use of caps with integrated electrodes may save time. In very young infants, it may be useful to place electrodes during sleep, as they are only awake and alert for a short time. If stimuli are designed sufficiently large, the eye-tracking calibration may be shorter or even omitted to save time (Kulke et al., 2015). There is a trade-off between the required accuracy and the loss of accuracy due to fewer trials until the infant gets fussy or falls asleep. The use of saline-based instead of gel-based electrodes may save time. Furthermore, the experiment should be designed in a flexible manner so that infants can feed and nap at any time.

As *data loss* is inherently greater when combining two methods, the number of conditions in an EEG–eye-tracking co-registration paradigm should be kept to a minimum in infant research. In gaze-contingent paradigms, it may be useful to program an alternative to gaze contingency. For example, if the eye-tracking signal is not sufficient to fulfil the gaze-contingency criteria, an additional loop may allow the manual starting of trials by the experimenter. This avoids EEG and overall data loss due to insufficient eye-tracking signals.

As the attrition rate is particularly high when combining EEG and eye-tracking, and recruitment can be challenging, the Many Babies model of international multi-lab collaborations may help increase sample sizes in co-registration data (Visser et al., 2022). This model initiates international collaborations in which several labs recruit and/or test infants, leading to larger sample sizes than could be recruited in single lab studies.

The analysis poses some additional challenges. Preprocessing pipelines for adult EEG data processing may need adjustments for infant data. For example, amplitudes of ERPs change with age, and therefore different artefact rejection procedures (Haresign et al., 2021) and thresholds based on medians for outlier detection in the EEG signal can be used (Kulke et al., 2015). The recently developed artefact detection pipelines can also be useful for EEG studies with newborns (Newborn EEG Artifact Removal (NEAR), Kumaravel et al., 2022), infants and children (Automated Pipeline for Infants Continuous EEG (APICE), Fló et al., 2022; Multiple Artifact Rejection Algorithm (MARA), Haresign et al., 2021; The Maryland analysis of developmental EEG (MADE), Debnath et al., 2020; The Harvard Automated Processing Pipeline for Electroencephalography (HAPPE), Gabard-Durnam et al., 2018; HAPPE In Low Electrode Electroencephalography (HAPPILEE), Lopez et al., 2022) and for developmental hyperscanning studies (dual EEG pipeline for developmental hyperscanning studies (DEEP), Kayhan et al., 2022).

### Open science

The replication crisis is a well-known problem in neuroscience (Nebe et al., 2023), particularly in EEG studies (Pavlov et al., 2021). Due to the larger data sets and increase in outcome variables, the potential for questionable research practices (Bailey, 2015; Banks et al., 2016; John et al., 2012; Nosek et al., 2015), particularly hypothesizing after the results are known (HARKing), “cherry-picking” of significant results (Andrade, 2021), or p-hacking (Hartgerink et al., 2016; Leggett et al., 2013) increases. When EEG and eye-tracking are combined, the resulting datasets are particularly large with numerous options for analyses so that the probability of a significant finding by chance increases compared to when just one measure is used. A solution to minimise false positive results due to questionable research practices is the preregistration of co-registration studies.

In infant research, preregistration of EEG studies is particularly difficult as amplitudes latencies of ERPs and frequency bands change with age (Barnet et al., 1980b; Bell & Fox, 1994; Gasser et al., 1988; Stroganova et al., 1999) and may be difficult to set in advance. However, Many Babies projects show that preregistration and even registered reports are possible in infant behavioural research (Visser et al., 2022). Although researchers often worry that preregistration may be too difficult, particularly with physiological measures, recent preregistrations show that preregistration of co-registered EEG and eye-tracking is possible in both adult (Kulke, 2019) and infant (Kulke, Ertuğrul, et al., 2021b) studies. Preregistration of co-registration studies should therefore become the norm in infant research and beyond.

## Conclusion

In summary, infant research has been a pioneering discipline in combining EEG with behavioural measures in natural situations, as infant behaviour cannot be controlled as easily as adult behaviour. The combination of EEG and eye-tracking in infants can be very useful in identifying the neural mechanisms underlying behaviour, particularly as behaviour and neural processing can diverge in real-world settings. Co-registered data can be used to improve data quality and test new hypotheses, but other challenges arise when studying infants, as pipelines were often developed for adults and as attrition rates can be high. Due to the large datasets, preregistrations and Open Science are important and possible. The combination of EEG and eye-tracking in infants can lead to novel insights about the development of attention in real-world interactions.
